# Should the scope of NIPT be limited by a ‘threshold of seriousness’?

**DOI:** 10.1038/s41431-024-01684-x

**Published:** 2024-08-16

**Authors:** Michelle Taylor-Sands, Molly Johnston, Catherine Mills

**Affiliations:** 1 Melbourne Law School, The University of Melbourne, Parkville, VIC Australia; 2 Murdoch Children’s Research Institute, Parkville, VIC Australia; 3 Monash Bioethics Centre, Monash University, Clayton, VIC Australia

**Keywords:** Health policy, Ethics

## Abstract

Non-invasive prenatal testing (NIPT) has the potential to screen for a wider range of genetic conditions than is currently possible at an early stage of pregnancy and with minimal risks. As such, there have been calls to apply a ‘threshold of seriousness’ to limit the scope of conditions being tested. This approach is based on concerns about society at large and the potential impact on specific groups within it. In this paper, we argue that limiting the scope of NIPT using the criterion of ‘seriousness’ is arbitrary, potentially stigmatises certain disabilities over others and fails to respect reproductive autonomy. We contend that concerns about expanded NIPT are more appropriately addressed by the provision of adequate information, counselling and consent procedures. We recommend a decision-making process that helps healthcare providers support prospective parents to make informed decisions about the nature and scope of NIPT screening based on their own values and social context. In addition to addressing concerns about expanded NIPT screening, this process would help clinicians to obtain legally valid consent and discharge their duty of care (including the duty to inform) in the prenatal context.

## Introduction

NIPT has been available in many countries since 2012. It was initially used to screen for the common foetal trisomies (such as Down syndrome). In recent years, commercial providers have begun to offer expanded NIPT screening, which may include rare autosomal aneuploidies, genome‐wide copy number anomalies, targeted microdeletions, sex chromosome aneuploidies or whole-genome sequencing [1]. Currently, in Australia and elsewhere, the delivery of NIPT is shaped by professional standards, clinical guidelines on prenatal testing, and common law principles relevant to the duty to inform and consent [2]. Several professional bodies recommend a permissive approach to NIPT to facilitate informed choice [3–5]. However, none of the professional bodies recommend routine expanded NIPT [3–5].

Expanded NIPT raises ‘societal concerns about what constitutes a “healthy pregnancy”, parental expectations of future children, equality, inclusivity and diversity’ [2]. Studies with past users of NIPT report mixed views on the expanding scope of NIPT. While many participants favour widening the scope of testing, views differ on whether the scope should be limited, and if so, how. Some users of NIPT express the view that there should be no limits to testing as having access to as much information as possible was perceived as a good thing [6]. Others were apprehensive of unrestricted testing, raising concerns about the potential for disability discrimination, a slippery slope to screening for ‘non-medical’ or ‘frivolous’ traits, and complicated decision-making [6, 7]. These concerns along with others, including ‘informal eugenics’ and the potential routinisation of screening, have been discussed extensively in the literature [8–11] and have led to calls for the scope of prenatal testing to be limited. Potential limits around prenatal testing were previously foreshadowed by Karpin and Savell, who asked whether a threshold of seriousness could be used as a ‘conceptual tool’ to limit excessive medical intervention in prenatal healthcare [12].

More recently, others around the world have called to limit the scope of NIPT screening to serious conditions to mitigate potential harm to society or specific groups within it [8, 10]. According to Thomas and others, expanded NIPT represents ‘a paradigm-shifting global technology—a mainstreaming of genomics’, which reignites previously unresolved debates about reproductive choice and responsibility [13]. Dondorp and colleagues argue that NIPT should only be offered for serious congenital conditions and childhood disorders [14]. The Nuffield Council on Bioethics recommends that NIPT ‘should not be used to test whether a foetus has a less significant medical condition or impairment’ [8]. As Brownsword notes, this raises questions about where this distinction should be drawn [15]. The Nuffield Council gives little clarification beyond describing certain conditions such as Triple X syndrome (associated with delayed learning, decreased muscle tone and kidney problems) and Van der Woude syndrome (associated with a cleft lip and palate) as not being significant [8]. Other commentators have debated how imposing a ‘seriousness’ threshold on the scope of conditions for which screening is offered might be used to limit access to NIPT. For instance, Bayefsy and Berkman propose a framework for limiting access to NIPT based on categories of genetic conditions that are perceived as differing in ‘seriousness’ according to clinical indicators such as suffering and early death [10]. In contrast, Kleiderman and others argue in favour of an adaptable framework utilising co-production of definitions of ‘serious’ that include personal, cultural and socio-economic factors [16].

Our paper explores whether there is an ethical basis for using the seriousness criterion to limit the scope of NIPT that is made available for pregnant people. We recognise that in practice, there are a variety of factors that impinge upon access to NIPT, including who might be able to afford an expanded test. Performance and clinical utility may also be relevant for limiting the scope of the test. Thus, we are not suggesting that seriousness is the only way to limit test scope in any given circumstance; rather, we are concerned with the question of whether seriousness should in principle be used as a reason to limit the test scope at all.

There is a need for robust ethical analysis and empirical evidence in determining whether limiting the scope of NIPT best addresses concerns about expanded NIPT. Given the potential for regulatory limits to negatively impact reproductive autonomy, it is important to ensure that individual choice is not eroded ‘wherever and whenever a voting majority can be assembled against them’ [17]. Our intention in this paper is not to examine the validity of concerns raised in relation to expanded screening but rather to evaluate whether a threshold of seriousness is a justified and workable response. In the following section, we critically analyse the ‘seriousness’ threshold, to argue that it is arbitrary, potentially stigmatises certain disabilities over others, and fails to respect reproductive autonomy. We recommend that concerns about the impact of expanded NIPT could be more appropriately addressed through improving practices in its clinical delivery that enable the scope of NIPT screening to be adapted for individuals on a case by case basis. In the final section, we outline a decision-making process that enables healthcare providers to support prospective parents to make informed decisions about the nature and scope of NIPT screening based on their own values and social context.

## Problems with imposing a ‘threshold of seriousness’

Drawing on ethical analysis and empirical data about how seriousness is understood by key stakeholders, we highlight three main arguments against imposing a ‘threshold of seriousness’ in NIPT screening.

Firstly, what constitutes a ‘serious condition’ varies for individuals, making it difficult to formulate an objective definition of a ‘serious condition’ that could be readily integrated into professional guidelines or other forms of regulation [16]. Wertz and Knoppers [18] surveyed 1,264 genetics professionals, asking them to provide examples of conditions they considered to be (1) lethal; (2) serious but not lethal; and (3) not serious. There was significant overlap between the categories; for example, 46% of the 267 conditions listed as ‘serious’ also appeared on the ’not serious’ list and 41% on the ’lethal’ list [18]. Reliance on related concepts such as ‘significant’ or ‘severe’ conditions is equally ambiguous. As Dive and others note in relation to genetic carrier screening, different stakeholders will vary in their views about ‘severity’ according to context, circumstances and personal views [19]. Barra and others similarly note that ‘severity’ in the healthcare context is an ‘essentially contested concept’ that incorporates needs, desires, suffering, social context and temporality [20].

There is also a lack of consensus in defining a ‘serious’ genetic condition in the context of expanded NIPT [21]. Our recent empirical work with health care professionals providing NIPT in Australia reveal wide-ranging understandings of seriousness or severity [22]. Factors that respondents incorporated into their conceptualisation of seriousness included: reductions in life expectancy, quality of life, and activities of daily living; impacts on others; and causing illness and/or disability [22]. Similar factors have been reported elsewhere in categorising the seriousness of a condition [23]. These results demonstrate that the concept of a ‘serious condition’ is multifaceted and likely reflects individual differences in understandings of health and disease.

Arguably, there is inherent vagueness in notions of seriousness or severity, based on the different features of a condition and how they affect the lives of individuals living with those conditions and those around them [24]. Variations in phenotype and penetrance influence understandings of seriousness, and not all people experience disability or disease in the same way. To date, attempts to rate seriousness or severity have prioritised biomedical information over qualitative aspects of the impact on individual lives (including carers) and been informed predominantly by clinical voices [16, 19]. Rubeis and Steger highlight the widespread misconceptions within society about the burden of disability [25]. There is a divergence between how ‘serious’ conditions are perceived and how they are experienced. Boardman and Clarke’s large mixed-methods study reported that people with ‘clinically serious’ conditions (as defined by Lazarin et al’s taxonomy [26]) frequently reported good health and the possibility of having a good quality of life – directly challenging assumptions about health outcomes often implicit in the classification of a serious condition [23].

Secondly, applying a ‘threshold of seriousness’ could potentially lead to the stigmatisation of certain disabilities. As noted above, how ‘seriousness’ is perceived is context-specific but drawing a line has implications for how different conditions are perceived. Dive and others note that the decision to include a particular condition in a screening programme is ‘not a neutral decision’ and ‘sends a message’ about the condition’s severity [19, 27]. Similarly, a report from Down Syndrome Australia highlights the impact that availability of screening may have on perceptions about quality of life experienced by those with the conditions screened, noting that ‘[b]ecause you can test for it, people must think it must be pretty bad’ [28]. This is problematic when a threshold of seriousness is externally imposed on prospective parents, particularly without adequate lived experience input. Ouellette argues that the ongoing challenges experienced by persons with disability regarding equality and inclusion are exacerbated by attempts to draw a line based on a threshold of seriousness [29].

Finally, further to whether it is possible to define what is ‘serious’, the question of *who* should be responsible for making that decision must also be addressed [10]. Users of NIPT are divided about on whom decision-making authority should fall – whether that be the State, healthcare professionals, or the user themselves [7]. Bayefsky and Berkmann [10] argue that decision-making authority should rest with medical practitioners; others argue that this itself raises problems for reproductive autonomy [30]. These problems are reinforced further when the state holds decision-making authority in this area, as this is overly reminiscent of eugenic policies and obscures the ethical import of the difference in individual views as outlined above.

Restricting the scope of NIPT screening by applying a ‘threshold of seriousness’ limits reproductive autonomy, and runs counter to current clinical guidelines for prenatal screening that prioritise a pregnant person’s access to information to make informed reproductive decisions. NIPT screening has both clinical and personal utility for prospective parents who may seek genetic screening for reassurance or to prepare for the future needs of a child [31]. Moreover, a prospective parent’s perception of seriousness does not necessarily predict interest in screening. A 2019 study by Sullivan and others [32] notes that ranking conditions by perceived seriousness does not always align with a pregnant person’s interest in screening for those conditions. The authors conclude that the motivations for screening are likely to vary and are influenced by external, non-clinical factors.

Given the issues associated with conceptualising and applying a threshold of seriousness, we conclude that limiting the scope of NIPT on this basis is unjustified and unworkable. In the next section, we propose an alternative approach for responding to the concerns associated with expanded NIPT.

## Alternative approach to address concerns

Instead of limiting the scope of NIPT on the basis of seriousness, we argue that concerns associated with expanded NIPT are better addressed through an approach that focuses on the *process of reproductive decision-making* and how it is best supported. This would involve the provision of adequate information, pre- and post-test counselling and consent. Importantly, the decision-making process should support prospective parents to understand the nature and limitations of NIPT, recognise the social aspects of ‘disability’, prepare for a positive result, and explore their own values and preferences as a precursor to genuinely autonomous decision-making. In this section, we outline an approach to counselling and consent that would enable healthcare providers to support prospective parents to make informed decisions about expanded NIPT. In addition to addressing the ethical concerns around expanded NIPT screening, we explain how this approach would help clinicians obtain legally valid consent and discharge their duty of care in the prenatal context. Whilst a detailed description of how our approach might be implemented in practice is beyond the scope of this paper, we outline a broad decision-making process and highlight some practical challenges, specifically in relation to counselling based on our recent research involving healthcare providers [1].

Our proposed approach to prenatal genetic counselling is based on the notion of ‘reproductive deliberation’ proposed by some of the authors, which ‘simultaneously recognizes the relationality of the counselling encounter and supports the decision making capacity and decisional responsibility of the pregnant person’ [33]. This approach aligns with a ‘process model’ for medical decision-making, whereby the discussions between a patient, healthcare provider and other relevant intimates constitute a *process* of informed consent [34]. This model acknowledges that a patient’s values and preferences are both elucidated and constituted through this process. Both reproductive deliberation and the process model for informed consent are influenced by relational autonomy, which recognises the sociorelational context within which reproductive decisions are made. Specifically, these approaches acknowledge that healthcare providers play a key role in sharing information, exploring motivations, counselling about implications, and clarifying values during the decision-making process.

Pre- and post-test counselling is central to our approach. Adequate pre-test counselling is important to avoid routine testing and address potential concerns about pressure to access a variety of tests [35]. In the context of genetic screening generally, Bunnik and others propose a ‘generic but differentiated’ approach to informed consent that provides individuals with sufficient but manageable information so that they can ‘opt out’ of receiving information they do not want [36]. McKinn and others recommend focused counselling for NIPT that provides prospective parents with both information about NIPT and values counselling [37]. This should include options that might arise out of expanded NIPT and how prospective parents may feel about the results.

It is important to acknowledge that while the importance of pre-test counselling has been asserted by many, there are several challenges to improving its provision. In our recent study, just over half of the healthcare providers surveyed considered pre-test counselling as moderately adequate and one fifth thought it was inadequate in preparing prospective parents for possible results [1]. Concerns were raised about time constraints on counselling, the quality of counselling given the variety of providers involved and their disciplinary backgrounds and the ability of patients to engage with and comprehend the information provided. These echo concerns reported elsewhere [38–40]. But challenges in improving pre-test counselling were also noted. While the professional societies and the Nuffield Council have made recommendations [3, 5, 8, 41], there is a lack of consensus on what information is relevant, and how it should be conveyed [42]. Notably, views of healthcare providers involved in providing counselling in Australia differ on what information is necessary to consent to the test [1]. Further, provider knowledge of the test, including what the test can screen for varies [1, 43], likely leading to inconsistent information provision. Given the amount of information to convey in pre-test counselling, we agree with others who argue that a relational approach that uses the values and preferences of the prospective parent(s) to guide information provision best serves reproductive autonomy [42]. However, this approach alone is insufficient in responding to the challenges discussed and needs to be supplemented to comprehensively address these in pre-test counselling for expanded NIPT. Furthering education on NIPT for healthcare providers, the development of materials (e.g. videos, decision-aids) to supplement counselling and support for the increasing time demands of comprehensive counselling would improve the pre-test counselling encounter and are likely necessary in the context of expanded NIPT [1, 41].

In the specific context of post-test prenatal genetic counselling, reproductive deliberation emphasises the importance of counsellors being responsive to patient requests for value judgments as opposed to always maintaining value-neutrality, as is the case with purely non-directive counselling [33]. As Warton et al highlight, post-test genetic counselling is:

*…crucial to support autonomy in the context of making complex and value-laden decisions about reproductive care following high-chance results from NIPT* [33].

However, in contrast to some shared decision-making models, decisional responsibility in reproductive deliberation remains with the pregnant person, thereby preserving their individual agency. By supporting the pregnant person to lead the counselling process and determine the information that is most relevant to them, reproductive deliberation focuses on *comprehension* of information rather than *comprehensiveness*. As Vears and others note in the context of genomic medicine, a balance must be struck between provision of sufficient information and overwhelming the patient [44]. Koplin and others suggest moving away from ‘fully informed consent’ toward ‘appropriately informed consent’ [45]. This approach aims to enhance genuinely informed decision-making in an area of medicine where the vast amount of information available can easily lead to overload and miscomprehension. Similarly, Bunnik’s layered approach to ‘individualised choice’ aims to promote reproductive choice without overwhelming prospective parents by providing individuals with sufficient information about categories of disease to enable them to determine the scope of screening that is relevant to them [36].

Although non-directive counselling may neither be attainable nor an effective approach to clinical practice [33], ‘non-directiveness’ can inform the counselling process for reproductive deliberation [33]. In the context of disability screening, inherent bias in language should be eliminated wherever possible. Despite recommendations to move to using neutral language when conveying results [28], our recent empirical work found that less than half of healthcare providers surveyed reported using neutral language (e.g. low/high probability or chance) to describe a negative or a positive result (36.5% and 44.4%, respectively) [1]. Further, several studies have shown that framing information in a positive or negative way can influence decision outcomes [46, 47]. As clinicians may be unaware of their directiveness [48], further education of counsellors around disability (including the social model of disability) could assist in revealing and addressing any internal bias [37].

The co-production of information tools, drawing on the knowledge of individuals with lived experience, would provide pregnant people with additional relevant information to help address the impact of unconscious bias, including but not necessarily limited to disability-bias. These information tools need not be specifically shared as part of the counselling process but could serve as adjunct decision-making aids for pregnant people to utilise as required to enhance comprehension, agency and informed consent. The use of adjunct tools would: (1) promote value-neutral provision of information that is not market-driven; (2) alleviate some of the time pressure during counselling [33]; and (3) empower pregnant people to manage the amount of information they receive based on individual values and preferences. There have been recent calls for further research into and development of alternative genetic counselling tools ‘such as telehealth, web-based educational videos, and computerized decision aids’ to supplement traditional genetic counselling services, specifically in response to expanded test panels [5].

In addition to promoting reproductive autonomy and addressing concerns around disability discrimination, complicated decision making and routinisation of screening, our approach would help clinicians to obtain legally valid consent and discharge their duty of care (including the duty to inform) in the prenatal context by providing relevant and comprehensible information to patients in an increasingly complex area of healthcare [2]. Given the increasing accuracy of NIPT and focus on reproductive autonomy, healthcare providers involved in antenatal care arguably have a legal duty to inform patients about the availability of NIPT and how they can access it in the private sector. Brownsword and Wale suggest the 2015 UK case of *Montgomery v Lanarkshire* [49] reinforces the importance of taking a pregnant person’s reproductive autonomy and consent seriously in reproductive settings and doctors may now be legally obliged to inform women about particular prenatal tests [15]. The duty on healthcare providers to inform patients about NIPT would logically extend to ‘the performance (and limitations), interpretation and communication of test results’ [8, 9] to ensure that patients have an adequate understanding of available treatment and care.

## Conclusion

Limiting the scope of NIPT based on a ‘threshold of seriousness’ is an unjustified and unworkable response to concerns raised about the impacts of expanded NIPT on society and specific groups within it. Specifically, a ‘seriousness’ threshold is arbitrary, potentially stigmatising and interferes with reproductive autonomy. Concerns about expanded NIPT are better addressed through the provision of appropriate and tailored information, counselling, and consent. The decision-making process we propose enables healthcare providers to assist prospective parents in making informed choices that align with their values and social context. Such an approach not only addresses key concerns but also supports clinicians in obtaining legally valid consent and fulfilling their duty of care in the prenatal context.
